# HIV Reservoirs in Brain parenchyma and Cerebrospinal Fluid During Suppressive ART: Same-Same or Very Different?

**DOI:** 10.1007/s11904-026-00790-3

**Published:** 2026-06-20

**Authors:** Thomas A. Angelovich, Janna Jamal Eddine, Emily K. Chalmers, Sarah J. Byrnes, Melissa J. Churchill

**Affiliations:** 1 ATRACT Leading Research Centre, School of Health and Biomedical Sciences, RMIT University, Melbourne, Australia; 2 Department of Infectious Diseases, The University of Melbourne at the Peter Doherty Institute for Infection and Immunity, Melbourne, Australia; 3 Life Sciences Discipline, Burnet Institute, Melbourne, Australia; 4 Departments of Microbiology and Medicine, Monash University, Melbourne, Australia

**Keywords:** Brain, Cerebrospinal fluid, HIV Reservoir, Neuroinflammation

## Abstract

**Purpose of Review:**

The central nervous system (CNS) remains a relatively understudied reservoir of HIV that may have significant implications for HIV cure and the pathogenesis of cognitive disorders that persist despite viral suppression with antiretroviral therapy (ART). This review will describe our current understanding of the nature, size and composition of the CNS reservoir and the possible contribution of viral persistence in the CNS on viral replication, neuroinflammation and cognitive disease.

**Recent Findings:**

Reservoirs of intact and defective HIV proviral DNA have recently been detected in both the brain parenchyma and cerebrospinal fluid (CSF) of ART-suppressed PWH. Moreover, viruses from these sites are transcriptionally and translationally active, with the potential to propagate infection ex vivo in the absence of ART. Ongoing viral persistence likely contributes to elevated risk of comorbid neurocognitive issues and measures of neuroinflammation support a pathological impact of HIV reservoirs on the brain. However, it is becoming clear that the sub-regions of the CNS including the brain parenchyma and CSF contain unique reservoir characteristics including cellular sources and reservoir dynamics which likely impart different effects on neuropathology.

**Summary:**

The CNS represents a heterogeneous reservoir of HIV with sub-regional differences in reservoir maintenance and cellular sources, highlighting the need to consider these nuances in HIV treatment, neuropathology and cure strategies.

## Introduction

The persistence of human immunodeficiency virus (HIV) in cellular and tissue reservoirs throughout the body is a major barrier to HIV cure and key contributor to comorbid diseases. Although sustained antiretroviral therapy (ART) use effectively suppresses HIV viremia in blood, approximately 20% of people with HIV (PWH) continue to experience significant neurocognitive impairment, including HIV-associated neurocognitive disorders (HAND) or HIV-associated brain injury (HABI) [1–5]. The mechanisms underlying the pathogenesis of cognitive disorders in PWH remain unclear but viral persistence within both peripheral tissues and the central nervous system (CNS) are likely key contributors. HIV reservoirs are pools of proviruses present during ART-suppression which may be sites of viral reactivation, ongoing neuroinflammation and potential viral rebound if ART is ceased. Therefore, understanding the role of reservoirs, particularly in the CNS, on viral reactivation and neuropathology is important to define improved treatment strategies.

The CNS is a collective term for the parenchyma of the brain, the brain stem and the cerebrospinal fluid (CSF). The brain parenchyma consists of the major tissue lobes of the brain, while the CSF is produced by the choroid plexus and encases the parenchyma within the skull to cushion it from impact, provide nutrient supply throughout the brain and remove waste bioproducts. Given the protected nature of the parenchyma and the inability to routinely biopsy the brain parenchyma itself, the CSF provides an accessible ‘window’ to the brain for both clinicians and researchers to quantify ongoing cellular activation, neurodegeneration and dysfunction and, importantly, the level of HIV viremia. However, questions have been raised recently as to whether the HIV reservoir in the CSF truly reflects that in the parenchyma or the peripheral blood or whether it alone is a unique reservoir of HIV. This review will provide a timely update on the dynamics of HIV persistence and activation in the CNS including within, and between, brain parenchyma and the CSF relative to peripheral cellular and tissue reservoirs. Furthermore, the implications of sub-regional reservoirs on cognitive performance, treatment and HIV cure strategies will be discussed.

## Evidence of HIV Reservoirs in the Brain Parenchyma During ART Suppression

**Fig. 1:**
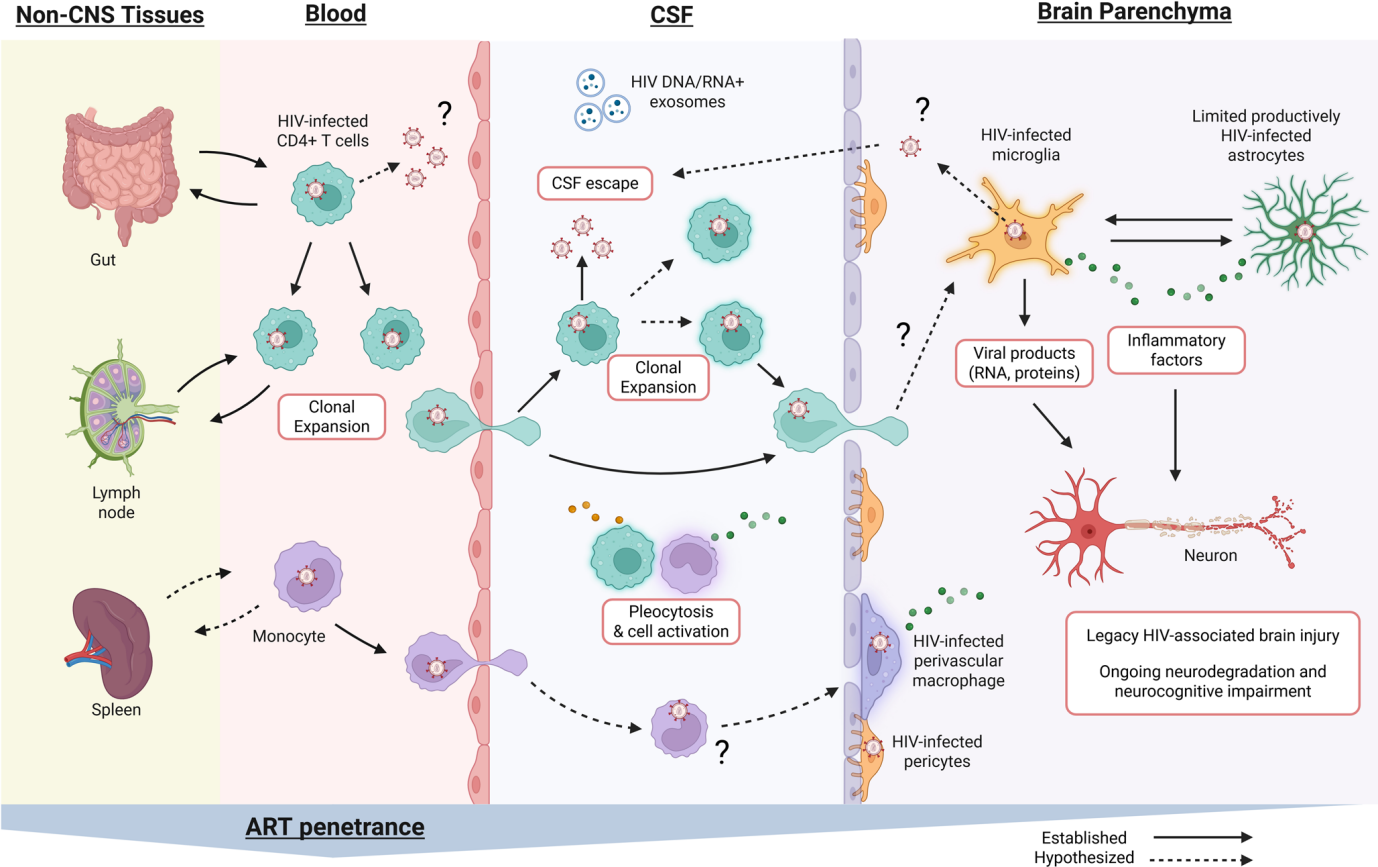
Proposed mechanism of HIV persistence and neuropathology in the CNS during ART suppression. Viral reservoirs in blood and peripheral tissues may traffic to the brain and cerebrospinal fluid (CSF). Reservoir expansion through clonal proliferation, and in some cases ongoing viral replication, supports persistence despite effective antiretroviral therapy (ART). Within the brain parenchyma, infection of microglia, perivascular macrophages, pericytes, and astrocytes promotes cellular activation and neuroinflammatory pathways that may contribute to neuronal injury. Persistent viral activation, and potentially replication, may occur in compartments with suboptimal ART penetration. CSF escape, defined by higher HIV RNA levels in CSF than in blood, may reflect local replication in the CSF or viral release from parenchymal brain reservoirs. Compartmentalisation is generally not observed between compartments in the presence of ART, however, clonal expansion in the CSF has been detected in some individuals. Solid arrows indicate established pathways; dotted arrows indicate hypothesized mechanisms. Created using BioRender

Studies in animal models support HIV entry into the CNS via the trafficking of circulating T cells and potentially monocytes through the CSF and across the blood brain barrier (BBB) which can then infect tissue resident cells [6–8] (Fig. 1). Specifically, HIV proviral DNA has been detected primarily in microglia and perivascular macrophages, while pericytes and astrocytes are also infected [9–16]. Until recently the size and nature of the HIV reservoir in the brain parenchyma during viral suppression with ART was unclear. However, a series of studies,

including our own, addressed this gap demonstrating that HIV DNA is present in autopsy parenchymal brain tissue from PWH who were durably ART-suppressed prior to death [11, 17, 18]. The level of HIV proviral DNA in brain tissue remained present at similar levels to those measured in viremic PWH, indicating that a stable reservoir of HIV persists despite long term ART [11, 18]. Sub-regional analysis throughout the brain found that the frontal cortex is a larger reservoir of HIV proviral DNA than either cerebellum or basal ganglia [11, 17]. The size of the reservoir in parenchymal brain tissue from ART-suppressed PWH is approximately 10–100 HIV DNA copies/10^6^ cells which is significantly smaller than that measured in peripheral cells. Importantly, intact proviral DNA assay (IPDA) measurements of brain parenchyma by ourselves [11, 17, 19] and Gabudza et al. [18] demonstrated the presence of both intact and defective HIV proviral DNA, indicating that a subpopulation of proviruses are intact and potentially replication competent. These observations are supported by near full length sequencing based studies identifying intact proviral genomes in basal ganglia tissue from PWH [20]. Similar to other reservoirs, the majority of HIV proviral DNA detected throughout the brain contain hypermutations and/or deletions, as measured by IPDA and sequence analysis, therefore reflecting a reservoir that is largely defective. Of course, characterization of the brain parenchyma is limited by the fact that brain tissue biopsies are almost never possible and therefore assessment of the brain tissue requires post-mortem samples or characterization in animal models.

## Cellular Reservoirs of HIV in Parenchymal Brain Tissue

While CD4 + T cells are considered the major reservoir of HIV in peripheral blood and tissues including lymph node and the gastrointestinal tract, HIV reservoirs in parenchymal brain tissues are comprised mainly of tissue resident cells. As mentioned, HIV DNA has been detected in microglia, perivascular macrophage, astrocytes and pericytes in the brain, with the myeloid reservoir considered to be the largest source of viral persistence [10, 13, 15, 16, 21, 22]. Whether infiltrating CD4 + T cells are also a significant parenchymal reservoir is unclear (Fig. 1). Studies of acute Simian-Human immunodeficiency virus (SHIV)-infection of non-human primate (NHP) models identified SHIV + T cells in the meninges during acute infection [6], supporting passaging of infected cells into the brain. However, a recent study in the simian immunodeficiency virus (SIV)-infected NHPs demonstrated that while treatment with anti-IL15 prior to SIV infection reduced T cell infiltration into the brain and microglial activation during acute infection, no difference in the frequency of HIV RNA+ cells was observed [23], demonstrating the ability of the reservoir in the brain to be established despite modulation of T cell trafficking into the brain. While these studies reflect reservoir establishment, it is important to note that whether resident T cell reservoirs exist in the brain during chronic ART-suppression also remains unclear. Further ex vivo human autopsy studies and controlled SIV/SHIV studies in NHPs will likely shed light on viral persistence in T cells in the brain.

## The Brain Parenchyma is a Transcriptionally and Translationally Active HIV Reservoir

HIV reservoirs in brain parenchyma have been traditionally considered transcriptionally and translationally quiescent with little evidence for the potential of viral rebound. However, technical advances in methods capable of quantifying and characterizing viral products both in vivo and ex vivo have determined that this is not the case. The level of SIV RNA+ cells in the brain of ART-suppressed NHPs remained present at similar levels to non-ART-treated animals [24]. Our laboratory used duplex HIV RNA digital PCR assays established by Yukl and colleagues [25, 26] to quantify the presence of HIV RNA transcripts in autopsy frontal lobe brain tissue of both non-virally suppressed and virally suppressed PWH [12]. Specifically, frontal cortex brain tissue from the majority of viremic PWH harboured HIV RNA transcripts reflecting initiation (TAR), elongation (Long-LTR, Pol) and completion (PolyA; *n* = 12/13) with 7/13 viremic PWH also demonstrating multiple splicing (Tat/Rev). In contrast, approximately 50% of ART-suppressed PWH tested had detectable levels of HIV PolyA transcripts in the frontal cortex (representing completion of HIV transcription in a subset of PWH) and only 2/10 ART-suppressed PWH had multiply spliced RNA which is highly predictive of viral protein expression. Therefore, total levels of HIV RNA transcripts and the proportion of transcripts reflecting completion of transcription (i.e. PolyA) and/or protein production were lower relative to viremic PWH with ART. CD68 + myeloid cells expressing p24 were also identified in brain tissue from these individuals, supporting that integrated HIV may reactivate and potentially replicate.

Importantly, findings from ART-suppressed PWH [15] and SIV-infected NHPs [15, 27] further showed that isolation of microglia from brain tissue and subsequent culture with susceptible cell lines resulted in viral outgrowth, demonstrating that replication competent viral genomes persist in brain parenchyma during viral suppression with ART. Specifically, a study utilizing the rapid autopsy Last Gift cohort isolated CD11b-expressing microglia from human brain tissue from 4 PWH where 2 individuals remained on therapy until death (< 30 HIV RNA copies/mL), 1 individual stopped therapy 18 days prior to death (275 HIV RNA copies/mL) and 1 individual stopped therapy 7 days before death (plasma viral load unknown) [15]. Post-isolation microglia were cultured for 14 days with ART prior to activation with the latency reversing agent SAHA and CM272 for 7 days prior to the addition of supernatant to microglia isolated from HIV-seronegative individuals [15]. The presence of HIV *de novo* infection and replication were measured by HIV RNA and p24 released into the supernatant, supporting ex vivo findings of a replication competent reservoir in myeloid cells from virally suppressed PWH. Whether viral replication in the brain can lead to infection of peripheral cells is unclear, but unlikely, with little evidence supporting reseeding of peripheral sites.

## HIV Persistence in the CSF

Similar to brain parenchyma and peripheral reservoirs, HIV can be detected in the CSF during both non-ART-suppressed and ART-suppressed HIV infection. HIV is readily detected in the CSF of non-ART-suppressed PWH, especially during acute infection and in cases of significant cognitive impairment including HIV-associated dementia [28]. Levels of HIV RNA in the CSF are approximately one log lower than those present in matched plasma samples from ART-naïve PWH [29]. However, cases of CSF discordance (i.e. CSF HIV RNA levels exceeding levels in plasma by > 0.5 log HIV RNA) and CSF escape (i.e. detectable levels of HIV RNA in the CSF, but undetectable in the plasma) also exist and are often linked to adverse cognitive outcomes. Therefore, monitoring of CSF viral load, especially in cases with symptoms indicative of neuropathology, remains an important clinical tool to inform underlying neuropathology and treatment approaches.

A series of studies over the past 10 years have demonstrated the presence of HIV RNA and HIV RNA+ cells in the CSF of PWH who were virally suppressed with ART and levels were often linked to adverse cognitive outcomes [30–37]. A study from the CHARTER cohort found that ART-suppressed PWH with detectable CSF HIV RNA often also had higher plasma viral load and pleocytosis in the CSF whilst displaying lower levels of CD4 + T cells and ART regimens with reduced CNS penetrance [33]. A large study of 1,264 ART-suppressed PWH found that 4.4% of individuals had detectable levels of HIV RNA in the CSF indicative of CSF viral escape [36]. While protease inhibitor use was an independent risk factor for CSF escape in this study [36], another study detected CSF escape in 28% of neurosymptomatic ART-suppressed PWH treated with NNTRI and NRTI-based regimens [37], indicating CSF escape is not solely linked to ART drug combination. Interestingly, while CSF viral escape was not associated with cognitive impairment in this study, others have reported that HIV CSF escape has been associated with a higher incidence of neuro-related symptoms including cognitive decline, confusion and headache relative to those without high CSF viral loads [38]. HIV RNA transcripts have also been detected in exosomes within the CNS of ART-suppressed PWH and that levels of RNA transcripts were present more frequently in PWH with worse cognitive scores [39]. Although the cellular sources of these exosomes were not defined, it is likely that they emanated from T cells within the CSF or plasma.

## Cellular Sources of HIV Persistence in the CSF

Unlike the brain parenchyma, cell associated HIV DNA and RNA is mainly restricted to CD4 + T cell populations, suggesting either passaging of infected cells into the CSF or clonal expansion within the CSF itself (Fig. 1). Utilizing novel RNA quantification tools, cell-associated HIV RNA transcripts have been detected in CD4 + T cells in the CSF of virally suppressed PWH [32]. Levels of cell-associated HIV RNA were associated with measures of brain injury in frontal white matter [34, 35], supporting a role for viral persistence in CSF in neuropathology. A study of 25 ART-suppressed PWH found that viruses from individuals with neurosymptomatic HIV CSF escape were mainly drug resistant with a mixture in viral diversity and propensity for CD4 + T cells [40]. Neurosymptomatic HIV CSF escape was associated with higher levels of neuroinflammation. However, a change in ART-regimen suppressed viral replication in CSF and reduced levels of neuroinflammation, demonstrating the potential to improve neuroinflammation by targeting viral persistence in the CNS.

Single cell RNAseq approaches have further identified that central memory CD4 + T cells in CSF were the major population to harbor HIV RNA and that levels of HIV RNA in T cells were higher in CSF than in PBMCs [31]. This was accompanied by altered CD8 + T cell phenotypes in CSF. Finally, the same group utilized TCR clonotyping to identify that the majority of T cell clones harboring HIV RNA in CSF were compartmentalized from the blood, supporting clonal expansion within the CSF. Importantly, a population of T cell clones were also present in both compartments, demonstrating trafficking between compartments [30]. Furthermore, a study from ART-virally suppressed PWH who underwent an analytical treatment interruption demonstrated the presence of R5 T cell tropic HIV viruses that is likely released from infected T cells in CSF [41]. Together, these findings demonstrate differences in the size and types of HIV reservoirs between the myeloid parenchymal reservoir of the brain and the predominately T cell reservoir of the CSF.

## Does Brain Parenchyma Harbor the Same HIV Variants as Those in CSF and Blood in ART-Suppressed PWH?

HIV exhibits differences in viral tropism between distinct tissue and cellular compartments in the absence of ART-suppression [42], likely reflecting the cell types present in each location. This is also true of viruses detected in parenchymal brain tissue, meninges, CSF and the blood with numerous studies have found differences in HIV viral *env* and LTR sequences between isolates from each regions supporting compartmentalization, especially during acute infection and HAD [43–47]. Examples of replicating viruses in the CSF of ART-naïve PWH were R5 T cell-tropic during acute infection [42]. Viral isolates from autopsy brain tissue are also functionally distinct from those in peripheral cells with polymorphisms present in the LTR that reduce responsiveness to latency reversing agents [48]. These findings support different cellular reservoirs of HIV between brain parenchyma and CSF, with the later more reflective of those found in peripheral blood.

Notably, while phylogenetic and genotypic studies from viremic PWH demonstrate compartments of distinct viruses, recent evidence suggests that this is not always the case in virally suppressed PWH. A study of 101 ART-suppressed PWH detected CSF escape (i.e. CSF viral load > 0.5 log copies relative to plasma) in 6 individuals and while env sequencing from 2 PWH identified likely transient passaging of T cell tropic viruses, one PWH had a virus more likely emanating from microglia or macrophage in the brain [49]. These findings support in rare cases possible low-level replication in the brain. Alternatively, evidence of passaging of clonally expanded cells from peripheral compartments to the CSF, and potentially parenchyma, have been reported. Clonal expansion in the CSF itself has also been reported [30]. A longitudinal study assessing intact full-length HIV env sequencing from multiple tissue sites from donors as part of the Last Gift cohort found similar viral sequences throughout multiple regions of the body, including the brain tissue [50]. A study of autopsy brain tissue from 63 PWH on ART obtained from the NNTC showed that HIV *gag* DNA was detected in 65.1% of PWH [51]. One PWH had the same HIV *env* sequence in brain and spleen, potentially indicating passaging of infected cells between compartments. Together, these findings suggest that persistent HIV in brain parenchyma and the CSF do not generally compartmentalize to peripheral cells and tissues in the presence of ART, however, can occur in a subset of individuals [30].

Viral persistence is maintained by multiple factors that may account for the lack in diversity seen. Firstly, while ART-suppression broadly limits new rounds of replication, it is possible that deep tissue microniches of cellular reservoirs with suboptimal levels of ART may contribute to low level viral replication both within parenchyma and the CSF. Secondly, the reservoir present in ART-suppressed PWH reflects the archival reservoir laid down during early infection and is more resistant to cell-mediated eradication and viral lytic mechanisms that are present in peripheral cells. Finally, clonally expanded reservoirs, particularly central memory CD4 + T cells in the periphery migrate and enter the CSF and potentially the brain parenchyma. However, an important caveat is the persistence of infected microglia in the brain which are long-lived and have low proliferative capacity. Therefore, further research is needed to characterize the sources of viral persistence in the brain during ART suppression.

## Considerations for the Role of Viral Reservoirs in the CNS on Neuropathology and Cognitive Impairment

HIV persistence in the CNS both directly and indirectly induces neuropathology and cognitive deficits in the brain of PWH in absence of ART. However, the specific contributions of HIV reservoirs both within, and external to the CNS, to neurocognitive disease remain ill-defined. Current data suggests that despite long-term viral suppression with ART, approximately 20% of PWH will develop cognitive disorders [1, 2] and that a subtle decline continues to affect virally suppressed PWH [3]. The classification of cognitive disorders in PWH is contentious with the previously accepted Frascati criteria of asymptomatic neurocognitive impairment, mild neurocognitive disease and HIV-associated dementia questioned as unnecessarily categorizing disease without symptomatic evidence [5, 52]. However, the approach remains widely supported indicating that further clarity is required to address the persistence of cognitive outcomes in asymptomatic PWH [4]. The mechanisms underlying cognitive disorders in PWH remain unclear and are likely to continue to be an issue with an aging population of PWH that must be recognised and considered in clinical management [53]. This is also important for PWH who are recently diagnosed as early ART-suppression is known to reduce HIV reservoir size and improve prognosis, therefore, likely also limiting HABI-induced neuropathology.

Cell-associated HIV DNA or RNA in the CNS has been associated with poorer cognitive performance and/or surrogate measures of neuroinflammation [32, 34], suggesting a specific impact of HIV viral reservoirs in the CNS on the pathogenesis of HIV cognitive impairment. Specifically, Spudich and colleagues demonstrated that ART-suppressed PWH with detectable HIV cell-associated (CA)-DNA in CSF had lower global deficit scores than those without detectable HIV CA-DNA [34]. Measures of HIV CA-RNA [32] and HIV transcripts detected in exosomes in CSF [39] also correlated with impaired measures of cognitive performance, suggesting that not only the presence, but the activity of HIV reservoirs, can impact cognition. However, these findings reflect detection/quantification of HIV DNA/RNA in the CSF, so whether these observations reflect the activity of the HIV reservoirs in parenchymal brain tissue or the periphery is unclear and needs to be considered.

Similar to other compartments in the body [54, 55], we and others have demonstrated that the bulk of the HIV reservoir in the brain consists of defective HIV proviral DNA which may impact cell activation in the brain [11, 17, 18]. The role of defective proviral DNA in the brain is unclear, but studies in peripheral cells and tissues have demonstrated that some defective proviruses are transcriptionally, and even translationally, competent producing RNA transcripts and viral proteins [56]. We have also demonstrated a relationship between the levels of both intact and 5’ defective proviral DNA in parenchymal brain tissue and measures of cellular activation, supporting an impact of both intact and defective proviral DNA. Whether this relationship impacts cognitive performance was not assessed by our study but is of key interest. However, it is likely that the presence and/or activity of both intact and defective reservoirs impact cognitive performance. Of course, factors external to the brain including cellular activation likely play a key role as we have demonstrated in the SIV NHP model that gut damage was associated with neuroinflammation [57]. Therefore, systemic inflammation should also be considered as a mechanism of neuropathology.

## Considerations for Therapeutically Targeting the HIV Reservoir in CNS

Current ART regimens are primarily aimed at limiting viral replication in the blood, with only specialist cases recommending screening of HIV RNA in the CSF as lumbar puncture can be uncomfortable and potentially unnecessary where no other symptoms indicate CNS complications. Therefore, measuring viral load in the CSF is not routine. As described, approximately 10–20% of ART-suppressed PWH exhibit CSF discordance with higher CSF viral load than present in plasma [49, 58–60]. CSF discordance and/or CSF escape is more prevalent in neurosymptomatic ART-suppressed PWH and those with ART regimens with poorer CNS penetration, however, still present in a subset of neuro-asymptomatic ART-suppressed PWH [58, 61]. ART intensification is not universally successful in reducing CSF viral load [62]. Therefore, continued monitoring of CNS reservoirs in the CSF are required to inform drug regimen. This is also required regarding the longer-term impacts of chronic HIV infection as a recent study demonstrated that detectable viral load was associated with possible accelerated age-related cognitive decline, however, this was not apparent in PWH with undetectable viral load, highlighting the need for early and effective viral suppression with ART [63]. The differing cellular composition of the HIV reservoir in the brain parenchyma relative to the CSF must also be considered in current treatment approaches as mainly myeloid cells in the parenchyma contain active HIV regardless of viral suppression in both the blood and CSF. Therefore, strategies aimed at reducing viral reactivation with cellular reservoirs in these compartments may be warranted in limiting viral persistence and cellular activation.

## Implications of HIV Persistence in Unique Sub-CNS Compartments for Cure

The presence of HIV proviral DNA in both parenchymal brain tissue and CSF cells poses major implication for HIV cure. Firstly, as a reservoir of replication competent HIV it is possible that HIV within CNS compartments may passage to peripheral cells and tissue, therefore, reseeding peripheral reservoirs. However, to date little evidence of reseeding of peripheral cells and tissue exists with only a study utilizing a humanized mouse model of infected astrocytes demonstrating viral escape from the CNS and infection of peripheral cells [14]. Alternatively, the presence of both intact and defective HIV proviral DNA in both CSF and parenchymal brain tissue likely contributes to cellular activation and potential neurocognitive impairment. Therefore, targeting these reservoirs is of interest for both the eradication of HIV and a functional cure. Current strategies aimed at HIV cure are often not specifically aimed at targeting the CNS which may be an oversight. Sampling the CNS is an involved process including lumbar puncture which study participants may be hesitant to complete. However, current strategies including anti-PD1 treatment, broadly neutralizing antibodies and chimeric antigen receptor therapy are likely to either have significant off-target effects in the brain or may not be able to penetrate the brain at all [64]. Therefore, the inclusion of CNS sub-studies aimed at establishing the impact of such approaches on the HIV CA-DNA or RNA in the CSF are required. Moreover, given the inability to sample parenchymal brain tissue well powered NHP studies may offer key preclinical insight to reduce risk of adverse effects on ART-suppressed PWH.

## Conclusions

Recent evidence has conclusively demonstrated that the CNS including both brain parenchyma and CSF remains a significant and stable reservoir of HIV that appears to not be reduced by long-term ART. While traditionally assessed as a combined site, subtle nuances in the cellular reservoirs between brain parenchyma and CSF exist which may reflect ongoing passaging of infected cells, clonal expansion or viral replication in niches of poor ART penetration. Together, these findings support the brain parenchyma and CSF as unique but related reservoirs of HIV. However, greater understanding of the mechanisms governing viral persistence across sub-compartments of the brain is required to inform treatment of both HIV viremia, but also neuroinflammation which contributes to neurocognitive issues impacting ART-suppressed PWH.

## Data Availability

No datasets were generated or analysed during the current study.
